# A case of eosinophilic cystitis coexisting of superficial bladder cancer, mimicking muscle‐invasive cancer

**DOI:** 10.1002/iju5.12594

**Published:** 2023-04-30

**Authors:** Yushi Suhara, Fumihiko Urabe, Masaki Hashimoto, Minoru Nakazono, Kosuke Iwatani, Shunsuke Tsuzuki, Shun Sato, Hiroyuki Takahashi, Takahiro Kimura

**Affiliations:** ^1^ Department of Urology The Jikei University School of Medicine Tokyo Japan; ^2^ Department of Pathology The Jikei University School of Medicine Tokyo Japan

**Keywords:** eosinophilic cystitis, mimicking invasive tumor, urothelial carcinoma

## Abstract

**Introduction:**

Here, we present a rare case of eosinophilic cystitis coexisting with bladder cancer, which appeared to be an invasive carcinoma on imaging.

**Case presentation:**

A 46‐year‐old man presented with urinary urgency. Computed tomography revealed an irregular and thickly enhanced bladder wall, which seemed to be invasive bladder cancer. Cystoscopy revealed a raspberry‐like mass lesion on the entire bladder circumference. Pathological diagnosis after transurethral resection was pathological T1 urothelial carcinoma. After a thorough discussion of treatment options, the patient elected to receive intravesical Bacillus Calmette‐Guérin. Three months after Bacillus Calmette‐Guérin administration, no residual disease was confirmed by transurethral biopsy, and no recurrence was observed over 2 years. As peripheral eosinophilia and submucosa eosinophil infiltration were identified, the patient was diagnosed with coexisting eosinophilic cystitis and urothelial carcinoma.

**Conclusion:**

Clinicians should consider the possibility of eosinophilic cystitis with superficial bladder cancer coexistence in patients who present with an irregular and thick bladder wall.

**Abbreviations & Acronyms**
AD: atopic dermatitis
BCG: Bacillus Calmette‐Guérin
DWI: diffusion‐weighted image
pT1: pathological T1
T1WI: T1‐weighted image
T2WI: T2‐weighted image

**Keynote message**

We encountered a rare case of eosinophilic cystitis coexisting with superficial urothelial carcinoma that mimicked muscle‐invasive cancer.

## Introduction

Eosinophilic cystitis is a rare bladder disease first described by Edwin Brown in 1960.^1^ Histological findings included eosinophil infiltration into the bladder wall. Here, we present a case of eosinophilic cystitis coexisting with superficial bladder carcinoma mimicking muscle‐invasive cancer.

## Case presentation

**Fig. 1:**
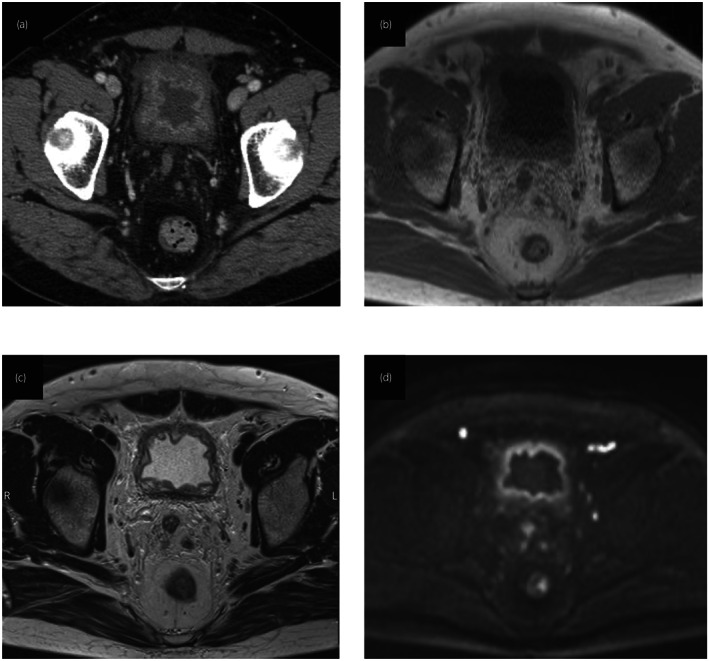
Enhanced computed tomography shows an irregular and thick enhanced bladder wall (a). The thickened bladder wall displayed homogeneous hyperintensity relative to the muscle on T1WI (b) and hypointensity on T2WI (c), and diffusion was slightly restricted on DWI (d).

A 46‐year‐old man with a month's history of urinary urgency and a jelly‐like substance in his urine was admitted to a previous hospital. His medical history included AD and childhood asthma. The urinalysis results showed microscopic hematuria (many/HPF) and pyuria (30–49/HPF), while the urine cytology test was negative. Enhanced computed tomography and magnetic resonance imaging demonstrated an irregular and thick enhanced bladder wall (Fig. 1a) with homogeneous hyperintensity relative to muscle on T1WI and hypointensity on T2WI; diffusion was slightly restricted on DWI (Fig. 1b,c). Based on these findings, the clinical stage was determined to be cT2N0M0 (VI‐RADS 4). Thus, his previous physician suspected invasive bladder cancer and performed transurethral resection of the bladder. Pathological diagnosis was a pT1 urothelial carcinoma. As imaging findings indicated the entire circumference of the muscle‐invasive bladder cancer, cystectomy was recommended. For radical surgery, the patient was referred to our hospital.

**Fig. 2:**
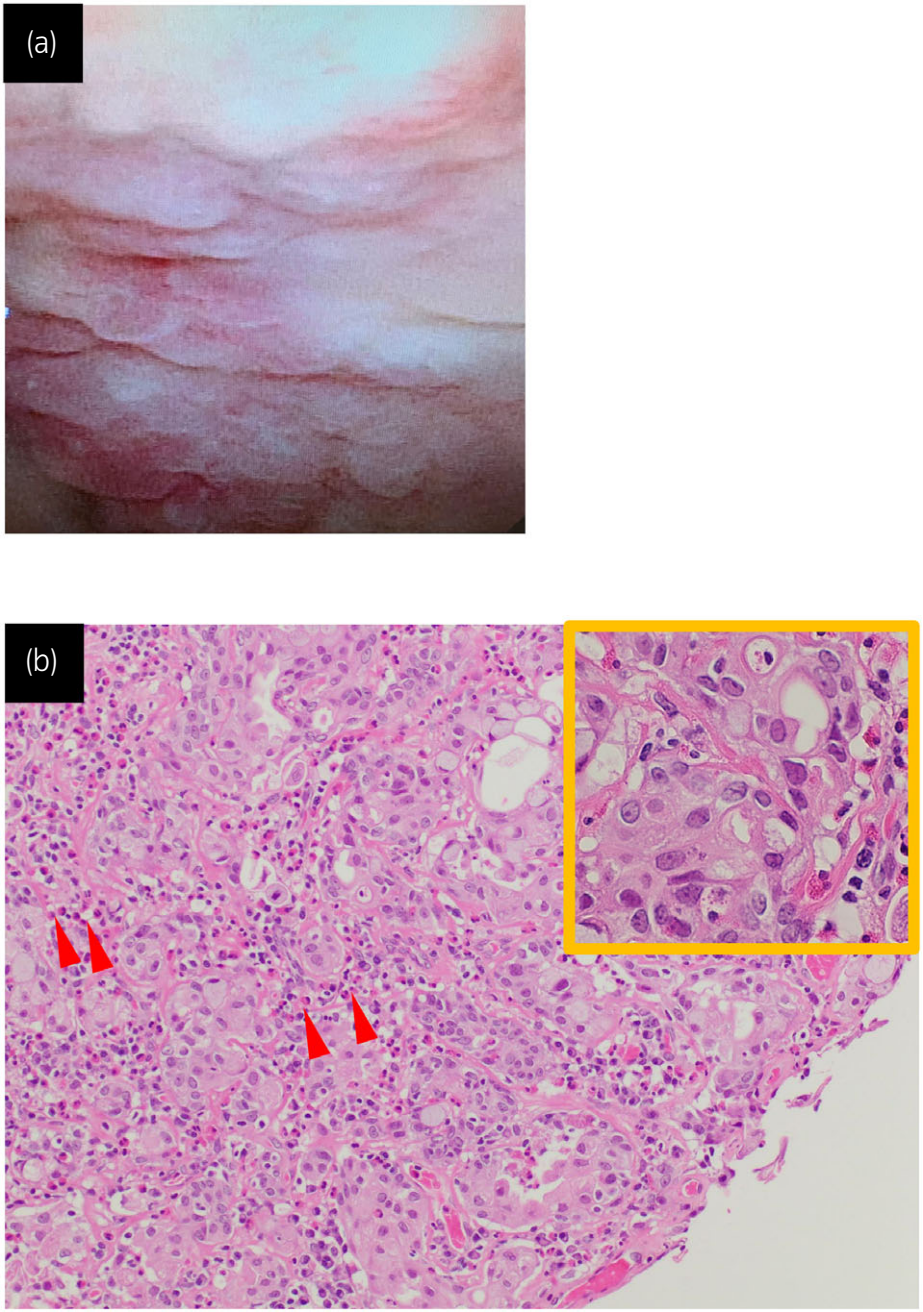
Cystoscopy revealed an erythematous mucosal lesion all over the bladder wall (a). Histopathologic examination of the specimen obtained by transurethral resection showed pT1 urothelial carcinoma and moderate infiltration of eosinophils (arrow head). (b). The tumor cells showed severe nuclear atypia on the high magnification. Hematoxylin and eosin (H&E) staining. Although a portion of the muscle layer was sampled, the tumor cells were found to only invade the subepithelial connective tissue, with no intramuscular invasion detected.

**Fig. 3:**
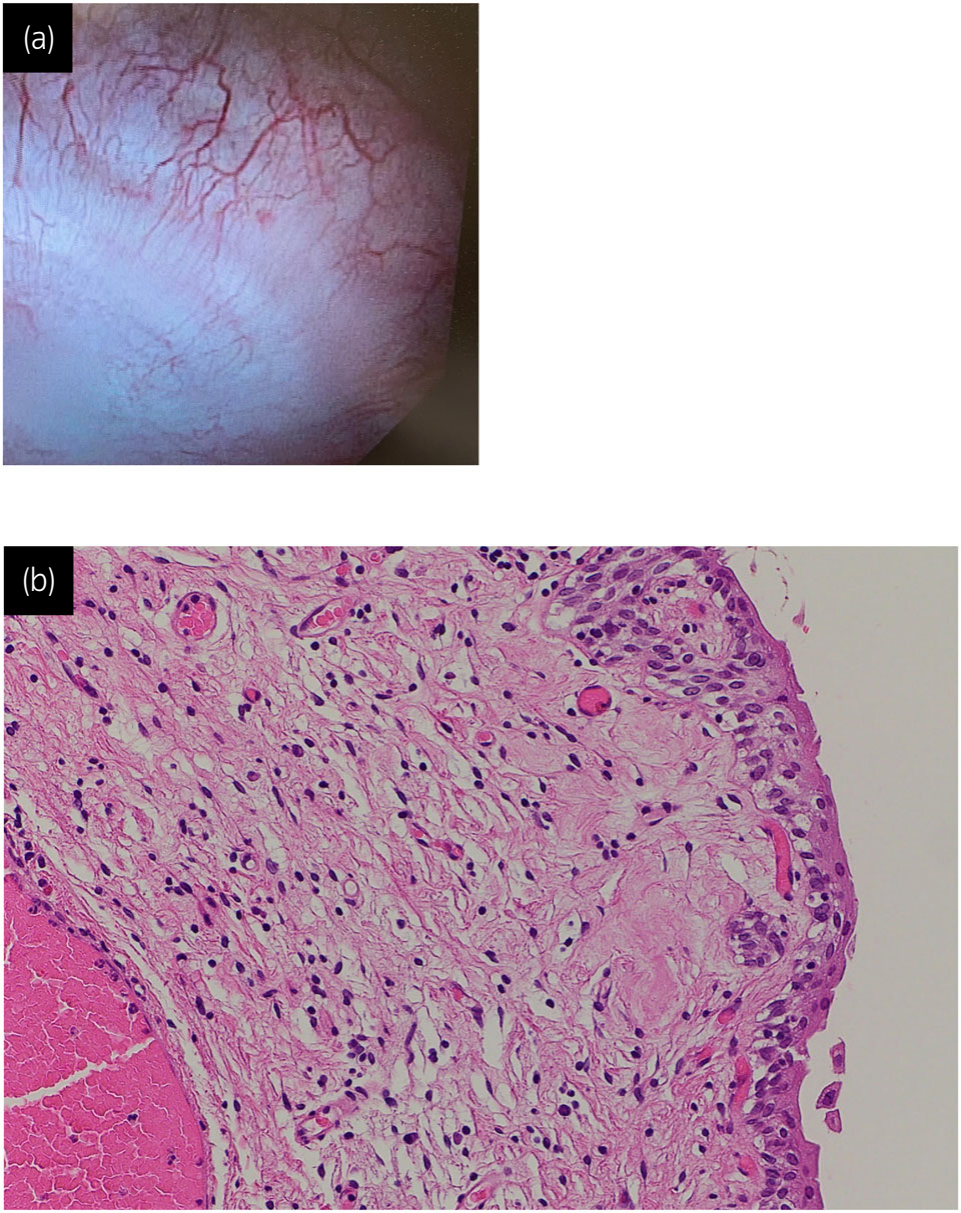
Cystoscopy on patient presentation showing no residual tumor (a). Histological finding of bladder revealed no residual tumor (b). Hematoxylin and eosin (H&E) staining.

**Fig. 4:**
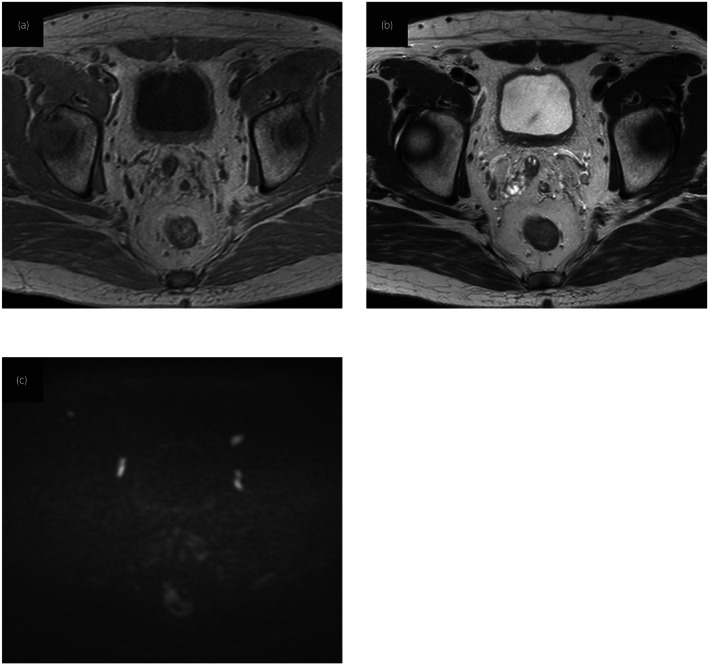
Magnetic resonance imaging after BCG therapy showing improvement. The bladder wall displayed homogeneous hyperintensity relative to the muscle on T1WI (a), hypointensity on T2WI (b), and diffusion was hardly restricted on DWI (c).

Cystoscopy revealed a raspberry‐like mass lesion on almost all sides of the bladder wall (Fig. 2a); however, urine cytology was also negative. These findings were irregular enough to confirm invasive bladder cancer; thus, we repeated transurethral resection. Pathological diagnosis was a pT1 urothelial carcinoma, which was observed in the entire circumference of the bladder (Fig. 2b). Additionally, there was no evidence of muscle layer invasion. After a thorough discussion of treatment options, the patient elected to undergo intravesical BCG rather than cystectomy. Intravesical BCG was administered once per week for 6 weeks. Subsequently, the patient's symptoms and imaging findings improved drastically (Fig. 3a). Three months after BCG treatment, cystoscopy suggested a complete response, and transurethral biopsy confirmed no residual disease (Fig. 3b). Following the BCG therapy, magnetic resonance imaging also demonstrated improvement in the thickening of bladder wall (Fig. 4). No recurrence was observed on follow‐up cystoscopy or urine cytology after 2 years.

Retrospectively, we present a patient with a history of AD and a high rate of peripheral eosinophils (12.3%). Bladder resection revealed moderate interstitial eosinophilic infiltration of the bladder subepithelial connective tissue (Fig. 2b). Based on these results, the patient was diagnosed with coexisting eosinophilic cystitis and superficial urothelial carcinoma.

## Discussion

Eosinophilic cystitis is a rare condition histologically characterized by eosinophil infiltration of the bladder wall. The major symptoms of eosinophilic cystitis are marked irritative lower urinary tract symptoms, such as dysuria, frequent micturition, and miction pain.^2^ Mass or thickening of the bladder wall is the main radiographic finding in eosinophilic cystitis, which sometimes resembles an invasive tumor.^3^, ^4^ The distinctive feature of our case is that not only radiographically but also pathologically, presence of urothelial carcinoma was confirmed twice by transurethral resection. The patient was initially diagnosed with invasive bladder cancer, and cystectomy was recommended. However, considering his history of AD and childhood asthma, high blood eosinophil count, and eosinophil infiltration in the bladder mucosa, the final diagnosis was eosinophilic cystitis combined with superficial bladder cancer. To the best of our knowledge, this is the second reported case of bladder cancer with eosinophilic cystitis.

Previous reports associated eosinophilic cystitis with a variety of infectious microbes, medications, allergens, urothelial carcinoma, and history of allergic diseases.^4^, ^5^ In the present case, the patient suffered from AD and childhood asthma, and his allergic predisposition would lead to eosinophilia and eosinophilic cystitis. Because of the immunological nature of the disease, corticosteroids have become the mainstay of treatment, especially in patients with the unresolved disease treated with anti‐inflammatory drugs.^6^ In the present study, pT1 urothelial carcinoma was first confirmed via transurethral bladder resection; therefore, intravesical BCG was administered. Although the precise mechanism of BCG efficacy in eosinophilic cystitis has not yet been elucidated, the complete response in bladder cancer has been investigated. As an association between urothelial carcinoma and eosinophilic cystitis has been reported,^5^ intravesical BCG removes the pathogen, which might improve cystoscopy findings. On the contrary, BCG itself is reported to be able to induce eosinophilic cystitis.^7^ Thus, although further accumulation of cases is required, cystectomy could be avoided, which would be pleasant for the patient. Kawai *et al*. previously reported a case of eosinophilic cystitis coexisting with bladder cancer in a patient who underwent a cystectomy.^8^ In their report, the preoperative diagnosis was invasive bladder cancer based on severe imaging findings; however, the final pathological diagnosis of the cystectomy sample was superficial bladder cancer. Thus, we recommend careful consideration of treatment strategies, including conservative therapy and cystectomy, if patients with bladder cancer are diagnosed with eosinophilic cystitis.

In conclusion, we encountered a rare case of eosinophilic cystitis coexisting with urothelial carcinoma that appeared to be a muscle‐invasive carcinoma on imaging. Intravesical BCG drastically improved imaging findings, and no residual tumor was confirmed by bladder biopsy. If the patient had eosinophilic cystitis, the imaging would appear as invasive bladder cancer. Although cystectomy might be chosen because of incurable urinary frequency and bladder pain, clinicians should keep in mind eosinophilic cystitis characteristics, and consider the proper treatment strategy for bladder cancer patients.

## Author contributions

Yushi Suhara: Conceptualization; data curation; writing – original draft. Fumihiko Urabe: Conceptualization; data curation; visualization; writing – original draft. Masaki Hashimoto: Conceptualization; writing – review and editing. Minoru Nakazono: Conceptualization; data curation; writing – original draft. Kosuke Iwatani: Conceptualization; data curation; writing – original draft. Shunsuke Tsuzuki: Conceptualization; writing – review and editing. Shun Sato: Conceptualization; writing – review and editing. Hiroyuki Takahashi: Conceptualization; writing – review and editing. Takahiro Kimura: Conceptualization; writing – review and editing.

## Conflict of interest

The authors declare no conflict of interest.

## Approval of the research protocol by an Institutional Reviewer Board

Not applicable.

## Informed consent

Consent to participate and for publication was acquired from the patient.

## Registry and the Registration No. of the study/trial

Not applicable.
